# Assembly of a Complete Mitogenome of *Chrysanthemum nankingense* Using Oxford Nanopore Long Reads and the Diversity and Evolution of Asteraceae Mitogenomes

**DOI:** 10.3390/genes9110547

**Published:** 2018-11-12

**Authors:** Shuaibin Wang, Qingwei Song, Shanshan Li, Zhigang Hu, Gangqiang Dong, Chi Song, Hongwen Huang, Yifei Liu

**Affiliations:** 1 Key Laboratory of Plant Resources Conservation and Sustainable Utilization, South China Botanical Garden, the Chinese Academy of Sciences, Guangzhou 510650, China; wsb1015@scbg.ac.cn (S.W.); songqingwei@scbg.ac.cn (Q.S.); lishanshan@scbg.ac.cn (S.L.); huanghw@scbg.ac.cn (H.H.); 2 Guangdong Provincial Key Laboratory of Applied Botany, Guangzhou 510650, China; 3 University of Chinese Academy of Sciences, Beijing 100049, China; 4 College of Pharmacy, Hubei University of Chinese Medicine, Wuhan 430065, China; zghu0608@163.com; 5 Amway (China) Botanical R&D Center, Wuxi 214115, China; tony.dong@amway.com; 6 Wuhan Benagen Tech Solutions Company Limited, Wuhan 430070, China; csong@icmm.ac.cn

**Keywords:** mitochondrial genome, *Chrysanthemum nankingense*, Asteraceae, Oxford Nanopore Technology, recombination, genome evolution

## Abstract

Diversity in structure and organization is one of the main features of angiosperm mitochondrial genomes (mitogenomes). The ultra-long reads of Oxford Nanopore Technology (ONT) provide an opportunity to obtain a complete mitogenome and investigate the structural variation in unprecedented detail. In this study, we compared mitogenome assembly methods using Illumina and/or ONT sequencing data and obtained the complete mitogenome (208 kb) of *Chrysanthemum nankingense* based on the hybrid assembly method. The mitogenome encoded 19 transfer RNA genes, three ribosomal RNA genes, and 34 protein-coding genes with 21 group II introns disrupting eight intron-contained genes. A total of seven medium repeats were related to homologous recombination at different frequencies as supported by the long ONT reads. Subsequently, we investigated the variations in gene content and constitution of 28 near-complete mitogenomes from Asteraceae. A total of six protein-coding genes were missing in all Asteraceae mitogenomes, while four other genes were not detected in some lineages. The core fragments (~88 kb) of the Asteraceae mitogenomes had a higher GC content (~46.7%) than the variable and specific fragments. The phylogenetic topology based on the core fragments of the Asteraceae mitogenomes was highly consistent with the topologies obtained from the corresponding plastid datasets. Our results highlighted the advantages of the complete assembly of the *C. nankingense* mitogenome and the investigation of its structural variation based on ONT sequencing data. Moreover, the method based on local collinear blocks of the mitogenomes could achieve the alignment of highly rearrangeable and variable plant mitogenomes as well as construct a robust phylogenetic topology.

## 1. Introduction

It has long been recognized that the structure of mitochondrial genomes (mitogenomes) is extremely variable among angiosperms, representing an important component of the whole genome’s diversity [1]. With the development of Next Generation Sequencing (NGS) technology, more than 150 mitogenomes of angiosperm species have been completely sequenced and assembled, but this is far less than the sequenced plastid genomes (plastomes > 2000 species; Organelle Genome Resources. Available online: https://www.ncbi.nlm.nih.gov/genome/browse#!/organelles/. Accessed on 21 October 2018). The genomic information from the mitogenomes of higher plants has been extensively used for the investigation of genomic evolution related to the gain/loss of genetic materials [2], phylogenetic relationships [3], nucleo-cytoplasmic interactions (e.g., cytoplasmic male sterility) [4], RNA editing [5], and genomic recombination and structural rearrangements [6]. However, the information of the diversity and evolution of mitogenomes within large angiosperm lineages remains limited.

One primary explanation for this lag is the chimerical variation in both the size and structure of angiosperm mitogenomes, which hinders their genome assembly and comparative analysis. Unlike the relatively stable genome size of plant plastomes (~150 kb), the mitogenome size of plants is highly variable, ranging from 66 kb in *Viscum scurruloideum* [7] to 11.3 M in *Silene conica* [8], and substantial size variation is even observed between closely related species (e.g., Cucurbitaceae, *Silene* and *Monsonia*) [8,9,10]. The gene contents of angiosperm mitogenomes also differ across species. Angiosperm ancestral mitogenome contains 41 variable protein genes (including 25 introns, five of which are trans-spliced), three constant ribosomal RNA (rRNA) genes, and insufficient transfer RNA (tRNA) genes necessary to recognize all codons [11]. During angiosperm evolution, mitochondrial genes have been widely lost or transferred to the nucleus, and ribosomal protein and succinate dehydrogenase subunit (*sdh*) genes are particularly frequently lost [12]. Remarkably, the *rps2* and *rps11* genes are lost in eudicots (Adams et al., 2002). The most abundant portion of the plant mitogenome is the intergenic regions [12], which include unknown functional sequences, promiscuous plastid and nuclear sequences derived by intracellular gene transfer (IGT) [13], as well as sequences derived from foreign organisms by horizontal gene transfer (HGT) [14]. In addition, indirect evidence from Southern blot and DNA sequencing reads pairs [7,15] suggests that the mitogenomes of angiosperms are dynamic as a consequence of recombination activities involving repeats [16].

Although diverse approaches have been used in plant mitogenome sequencing projects, the complete genome assembly using only NGS short reads remains a challenge [17], due to the lengths of the repeats or plastid-derived fragments in the mitogenome being generally larger than the read lengths (<400 bp) from NGS platforms. Sequencing from well-purified mitochondria can reduce the assembly complexity associated with the contamination of both nuclear and plastid sequencing reads [9,18], but the purification of plant mitochondria from diverse tissues is usually labor intensive and time consuming. Updated single-molecule sequencers with exceptional long-read capabilities have ushered in a new era of genome sequencing where the read lengths exceed those of the genomic repeats. In particular, Oxford Nanopore Technology (ONT) offers ultra-long reads of up to 2 Mb (https://nanoporetech.com/), which should easily span any repeats and plastid-derived regions, and should even theoretically cover whole plastomes (~150 kb) and the majority of whole mitogenomes (200–800 kb). Many successful applications of ONT in genome assembly have been reported, including small bacterial genomes [19] and large higher plant genomes [20]. To our knowledge, however, the application of long reads derived from ONT to assemble complete plant mitogenomes has not been reported. Comparatively, some studies have assembled the complete mitogenome from long sequencing reads from the Pacific Biosciences (PacBio) sequencing technologies [21,22].

As one of the largest angiosperm families, Asteraceae comprises 24,000–35,000 species, representing ~10% of all angiosperm species [23]. Complete mitogenome sequences in this family are only currently available for two species, *Helianthus annuus* [24,25] and *Diplostephium hartwegii* (NCBI Reference Sequence: NC_034354). Thus, mitochondrial information for Asteraceae is quite limited. *Chrysanthemum nankingense* (Previously thought to be a variety of *C. indicum*) belongs to Asteraceae and has important medicinal and ornamental value [26]. Despite recent progress in the nuclear genomic sequencing of *C. nankingense* species [27], mitochondrial information is still lacking. In the present study, we aimed to explore the effectiveness of complete mitogenome recovery in *C. nankingense* and investigate repeat-mediated homologous recombination based on the ONT long sequencing reads. Additionally, we retrieved public genomic Illumina sequencing datasets to assemble draft mitogenomes of 26 Asteraceae species and to investigate the diversity and evolution of the Asteraceae mitogenomes. Our results revealed significant improvements to complete mitogenome assembly using ONT long reads and also identified clear repeat-mediated homologous recombination within the *C. nankingense* mitogenome. Further comparative analysis between Asteraceae mitogenomes showed wide variation in both genomic content and structure, which represents a critical subset among the diversity presented across Asteraceae species. Last, we compared the phylogenetic topologies based on different datasets from core fragments and protein-coding regions of 28 Asteraceae mitogenomes and plastomes and provided a method utilizing local collinear blocks of mitogenomes to reconstruct robust phylogenetic relationships.

## 2. Materials and Methods

### 2.1. Materials and Sequencing Data

We obtained plant material of *C. nankingense* from Nanjing, Jiangsu Province of China. The cetyltrimethylammonium bromide (CTAB) method [28] was used to extract genomic DNA from fresh leaf tissue, and then sequencing was performed on both the HiSeq 2000 and ONT sequencing platforms. Trimmomatic v0.38 [29] was used to remove low quality reads from the Illumina data with the following parameters: LEADING:3 TRAILING:3 SLIDINGWINDOW:4:15 HEADCROP:8 MINLEN:36. Nanofilt v2.2.0 [30] was used to remove reads shorter than 500 bp or with an average quality score <7 from the base-called ONT sequencing data.

### 2.2. Assembly of the Mitogenome Using Three Different Strategies

Unlike the plastome, a strategy based on closely related mitogenomes as a reference is not suitable for the assembly of plant mitogenomes because of the variable size and dynamic structure among closely related species. The full assembly of the plant mitogenome from only Illumina sequencing data thus remains a challenge. In order to investigate the optimal assembly strategy, we assembled the *C. nankingense* mitogenome with three different methods: An Illumina-only method using only the Illumina short reads for assembly; a hybrid method using both the Illumina short reads and ONT sequencing reads; and an ONT-only method only using the filtered mitochondrial ONT sequencing reads for assembly. The data used for each method are shown in Table S1.

For the assembly of the Illumina-only method, two software programs, SPAdes v3.8.1 [31] and SOAPdenovo2 v2.40 [32], were used to assemble the mitogenome with different *k*-mer values (33, 55, 77, 99, and 127). For the hybrid assembly from the Illumina and ONT data, only SPAdes [31] was used with different *k*-mer values. For the assembly using the ONT-only assembly method, the mitochondrial long reads were firstly filtered from the total ONT sequencing data using BWA v0.7.16 [33] and SAMtools v1.9 [34] using the contig of hybrid assembly as the reference. Then, an ONT-only assembly was generated using Canu v1.7 [35]. Subsequently, the mitochondrial candidate contigs were identified by BLASTN [36] searching of each assembly against the *H. annuus* mitogenome [24]. Pilon v1.22 [37] was used to improve the assemblies. Last, QUAST v5.0.0 [38] was used to compare the results.

### 2.3. Annotation of the Mitogenome

The protein-coding genes of the mitogenome were identified by BLASTN searches against the collected plant mitochondrial protein-coding gene database, which was created using the command “makeblastdb” [36]. The start/stop codons and the exon-intron boundaries of the genes were adjusted manually. The tRNA and rRNA genes were identified using tRNAscan-SE v1.21 [39] and RNAmmer 1.2 Server [40], respectively. The open reading frames (ORFs; >300 bp) and repeats (>95% identity and >50 bp in length) were identified using the plugins in UGENE v1.30.1 [41]. Moreover, the plastid-derived sequences in the *C. nankingense* mitogenome were identified by using the BLASTN [36] searching the mitogenome against its chloroplast genome (plastome), with an E-value of 1e-50 and a minimum length of 100 bp as thresholds. Plastid-derived genes were annotated according to the annotation of the plastome. We adjusted the mitogenome, starting from the *atp1* genes, and visualized the circular physical map of the mitogenome using Circos v0.69 [42].

### 2.4. Identification of Repeat-Mediated Recombination

To examine the recombinational activity of repeats, we mapped the ONT reads to the different putative mitochondrial conformations, which were adjusted manually based on different repeat-mediated recombinations. Then, we calculated the recombinational frequency of the ONT reads that supported the alternative conformation (AC) of each repeat pair and the master conformation (MC). Last, we aligned these long reads supporting different conformations to show the recombination using NUCmer [43].

### 2.5. Assemblies and Annotations of Asteraceae Organelle Draft Genomes

To investigate the variations in gene content and constitution of the Asteraceae mitogenomes and evaluate the usefulness of mitochondrial DNA for phylogenetic analysis, we assembled near-complete organelle genomes from 26 Asteraceae species and one Calyceraceae species, *Nastanthus patagonicus,* as an outgroup (Table S2). The corresponding Illumina sequencing data of the genomic DNA were retrieved from the NCBI SRA site (https://www.ncbi.nlm.nih.gov/sra) using SRA-Tools (Table S2). The organelle draft genomes were de novo assembled based on the optimal assembly method of the Illumina-only data mentioned above (see discussion section). The organelle assemblies of each species were performed using SPAdes [31] with corresponding *k*-mer values (Table S2). The candidate mitochondrial contigs and plastid contigs of each species were filtered from the corresponding assembly result by using BLASTN [36] searches against the *C. nankingense* mitogenome and plastome, respectively. The filtered contigs were further polished using Pilon v1.22 [37].

We employed a two-step strategy to identify the presence/absence of mitochondrial genes. Firstly, we used BLASTN [36] searching the mitochondrial candidate contigs against the collected plant mitochondrial protein-coding genes database to validate the presence/absence of mitochondrial genes. Secondly, the uncertain genes with fragmentary matches were further analyzed by mapping the raw sequencing data to the corresponding gene sequence. The presence/absence of a gene was assessed according to the differences of sequencing depth of this gene and the average sequencing depth of the whole mitogenome. The gene matrix heat map was generated using the “pheatmap” package in R [44,45].

### 2.6. Analysis of Asteraceae Mitogenomes Constitution

To investigate the variation of Asteraceae mitogenomes constitution, multiple whole genome alignment of the mitogenomes was performed using Mugsy [46]. The Asteraceae mitogenomes were parsed to three different types of fragments (core fragments, variable fragments and specific fragments) from the output of Mugsy using our customized shell scripts. The core fragments were shared by all the 28 Asteraceae mitogenomes. The specific fragments were unique to a single mitogenome. And the variable fragments were shared by a few mitogenomes. The size accumulation curves of the pan mitogenome and core mitogenome of 28 Asteraceae species were obtained using PanGP v1.0.1 [47] following multiple whole genome alignment analysis by random sampling of up to 500 replicates for each group. The size information of core mitogenome was the accumulation of sizes of the core fragments of each group. The size information of pan mitogenome was the accumulation of sizes of core fragments, variable fragments and specific fragments, of which the sizes of homologous sequences among core and variable fragments will count only once.

### 2.7. Phylogenetic Analysis of Asteraceae Organelle Genomes

Phylogenetic analyses from 28 Asteraceae species and one outgroup, *N. patagonicus,* were performed using four datasets: (a) local collinear blocks (LCBs) of mitogenomes; (b) LCBs of plastomes; (c) protein-coding regions of mitogenomes; (d) protein-coding regions of plastomes. The protein-coding regions were extracted from the final assemblies using BLASTN [36] and searched against the gene databases of plant plastomes and mitogenomes. The LCBs of the mitogenomes and plastomes were identified using Mugsy [46] with default parameters. These datasets were aligned using MAFFT v7.310 [48]. The poorly aligned positions and divergent regions of the alignments were eliminated using Gblocks v0.91b [49] with the following parameters: −t = c − b5 = h − b4 = 10. A supermatrix was constructed using SequenceMatrix v1.7.8 [50] to concatenate different genes or LCBs. The Akaike information criterion (AIC) was used in jModelTest v2.13 [51] to select models of character evolution in each supermatrix. A maximum-likelihood (ML)-based phylogenetic tree of the concatenated supermatrix was constructed using RAxML v8.2.10 [52] with 1000 bootstraps. The parsimony-informative characters were analyzed using MEGA7 [53].

### 2.8. Data Accessibility

The mitogenome of *C. nankingense* have been deposited in the Genbank database under accession MH716014. The assembled plastome and mitogenomic fragments and the detailed source codes used for the analysis are provided at https://github.com/wangshuaibin1015/Chrysanthemum-nankingense-mitogenome.

## 3. Results

### 3.1. Comparison of Different Assembly Strategies

**Figure 1:**
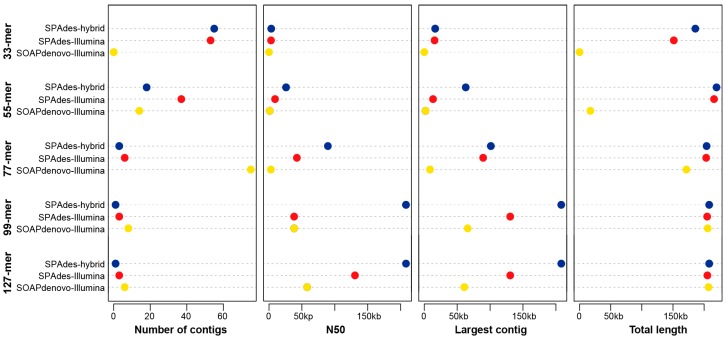
A comparison of the assemblies based on different strategies and datasets. SPAdes-hybrid indicates that the assemblies were generated using both Oxford Nanopore Technology (ONT) and Illumina data. SPAdes-Illumina and SOAPdenovo-Illumina indicate that the assemblies were generated using Illumina-only data. kb: kilobase; N50: the N50 was defined as the sequence length of the shortest contig at 50% of the total genome length.

**Figure 2:**
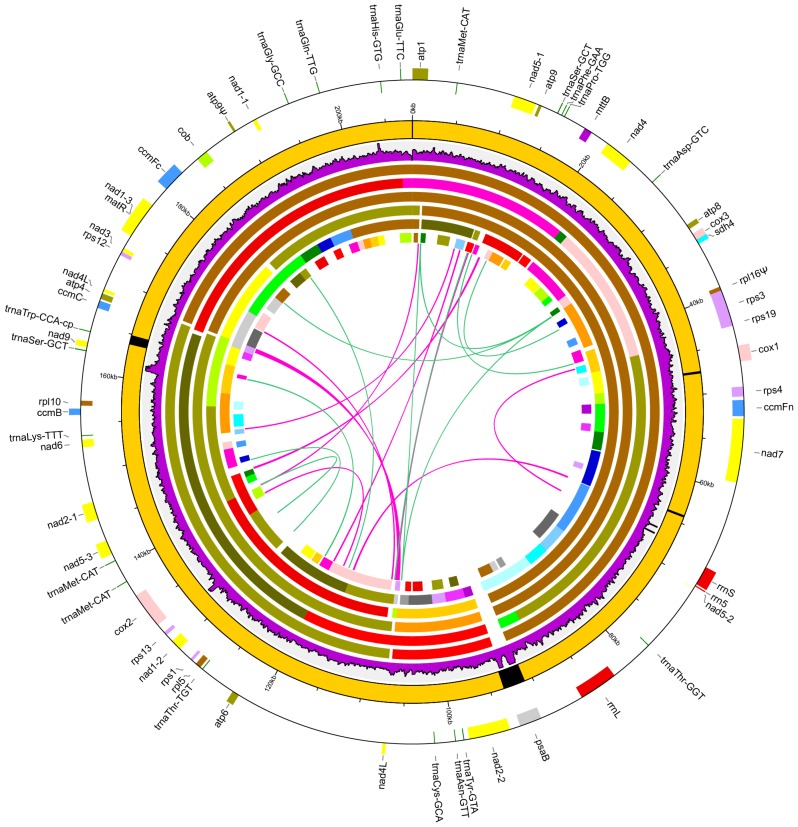
Maps of the *Chrysanthemum nankingense* mitogenome. Peripheral gene blocks shown on the outside and inside of the circle were transcribed clockwise and counter-clockwise, respectively. The inner eight layered circles indicate the main circle molecular structures, the sequencing depth rate of the Illumina data along the mitogenome and six assemblies from the Illumina-only data with different *k*-mer values. The black in the main circle indicates the plastid-derived fragments in the mitogenome. The six assemblies from 127-mer, 127-mer, 99-mer, 77-mer, 55-mer, and 33-mer are shown from outside-to-inside. The mitochondrial candidate contigs are distinguished using different colors. The repeats are shown in the innermost circle. Repeats of >100 bp are indicated by connecting red bands. Repeats of <100 bp are indicated by connecting green lines.

A total of 40 million Illumina sequencing reads with a mean length of 150 bp and 1.36 million ONT reads with a mean length of 20.3 kb were generated, which comprised one part of the whole genome sequencing project of *C. nankingense* [27]. We compared the results derived from the Illumina-only assemblies and the hybrid assemblies based on different *k*-mer values using both SOAPdenovo2 and SPAdes (Figure 1). For the Illumina-only assemblies, the assemblies from SPAdes were always better than those from SOAPdenovo2, showing longer size and fewer number of the total candidate contigs and larger size of the largest contig (Figure 1), despite being more computationally intensive. Furthermore, with the increase in *k*-mer value from 33 to 127, the assemblies were noticeably improved, with decreased numbers and increased lengths of the candidate contigs (Figure 1). The best assembly based on only the Illumina data from the 127-mer failed to obtain a single whole mitogenome but produced three candidate contigs (the largest one was 130,635 bp in length) with a total size of ~208 kb. Notably, despite the different levels of the assemblies in terms of the number of contigs, the N50, and the largest contig, little change was observed in the total size of each assembly from the Illumina-only dataset, with relatively large *k*-mers obtained using SPAdes (Figure 1), which provided an optimal method for recovering the near-complete mitogenome from the accessible Illumina sequencing data of other angiosperms. Comparatively, all the assemblies based on the hybrid assembly method showed a significant improvement (Figure 1). Particularly, only one candidate contig (~208 kb) was identified from the hybrid assembly with the 99-mer and 127-mer. This contig was recognized as the whole mitogenome of *C. nankingense* after cyclization by end repeats. After further polishing by repairing one mismatch and 12 gaps/insertions, we finally generated a 208,097 bp circular molecular map (Figure 2) with read depths of 51× and 12× in the Illumina and ONT sequencing data, respectively. For the ONT-only assembly, a total of 116,898 reads with a mean length of 30 kb were filtered from all ONT sequencing data and assembled using Canu. Then, only one 232,074 bp contig was filtered from the assembly with a pair of 25,700 bp repeats in the ends. Compared with our polished hybrid assembly, the ONT-only assembly revealed 1251 single base deletions and 40 mismatches, which suggested that the result from the ONT-only assembly required further polishing using the Illumina sequencing reads. Our subsequent analysis of the mitogenome was based on the polished hybrid assembly.

### 3.2. General Features of the Chrysanthemum nankingense Mitogenome

The *C. nankingense* mitogenome encoded a total of 55 functional genes (Figure 2 and Table S3), including 33 protein genes (two *nad4L* genes included), three rRNA genes (*rrn5*, *rrn18* and *rrn26*), and 19 tRNA genes. Moreover, a total of 21 group II introns (15 *cis*-spliced and 6 *trans*-spliced) were detected, in which eight intron-containing genes were identified (*ccmFc*, *cox2*, *nad1*, *nad2*, *nad4*, *nad5*, *nad7*, and *rps3*) (Table S3). Furthermore, the *rpl16* gene and a duplication of the *atp9* gene were detected as pseudogenes for the premature termination codon mutations. Compared with the ancestral gene content of angiosperms [18], seven mitochondrial genes were missing from the *C. nankingense* mitogenome, including the *rpl2*, *rps2*, *rps7*, *rps10*, *rps11*, *rps14*, and *shd3* genes. In comparison to the closely related *H. annuus* mitogenome, the *C. nankingense* mitogenome contains an additional *rps1* gene (Table S3). The 19 tRNA genes recognize a total of 15 amino acids (Asn, Asp, Cys, Gln, Glu, Gly, His, Lys, Met, Phe, Pro, Ser, Thr, Tyr, and Trp). The coding exon regions were a total length of 31,922 bp, representing 15.34% of the mitogenome (Table S4). The rRNA and tRNA genes were a total length of 5963 bp and 1440 bp, accounting for 2.87% and 0.69% of the mitogenome, respectively. All of these functional regions were larger than those identified in the *H. annuus* mitogenome, even though the size of the *H. annuus* mitogenome was much larger than that of *C. nankingense* (Table S4). The overall GC content of the *C. nankingense* mitogenome (45.41%) was slightly higher than that of the *H. annuus* mitogenome (45.05%, Table S4).

The intergenic spacer regions represented the largest part of the *C. nankingense* mitogenome, with a total length of 168,772 bp accounting for 81.10% of the mitogenome. A total of four plastid-derived sequences were identified throughout the *C. nankingense* mitogenome, ranging from 246 to 2555 bp in length (Table S5). The total length of the plastid-derived sequences was 4122 bp, representing 1.98% of the mitogenome. Only one plastid-derived protein-coding gene, *psaB* gene coding the A2 subunit of photosystem I in plastome, with an intact ORF was identified as a pseudogene. In addition, a total of 30 ORFs were identified in the mitogenome intergenic regions (Table S6). The majority of the ORFs were <1 kb, with the exception of one ORF with a length of 2064 bp encoding a DNA polymerase-like gene, which was absent from the *H. annuus* mitogenome (Table S3).

### 3.3. Repeats and Homologous Recombination

Repeats analysis of the *C. nankingense* mitogenome revealed 22 DNA repeats with a total length of 5372 bp, ranging from 50 to 681 bp (Table S7). These repeats constitute 2.58% of the genome, including 1.84% intermediate (100–1000 bp) and 0.74% small (<100 bp) repeats, while no large repeats (>1 kb) were detected (Table S8). Interestingly, the intact *nad4L* gene is contained in the largest repeat (681 bp), resulting in two copies of this gene present in the mitogenome (Figure 2).

**Figure 3:**
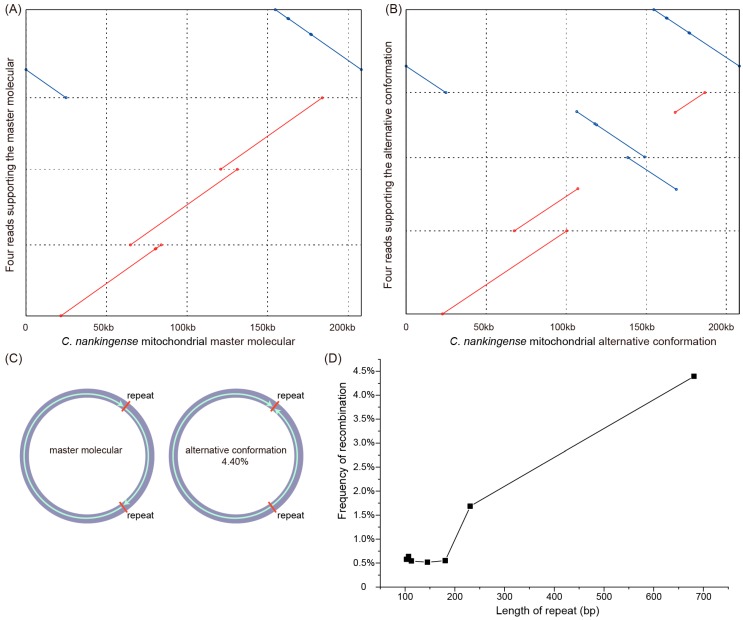
The master conformation and alternative conformation of the *C. nankingense* mitogenome. (**A**) Four ONT long reads support the master conformation (MC) of the *C. nankingense* mitogenome. (**B**) Four ONT long reads support the AC of the *C. nankingense* mitogenome. (**C**) The isomeric forms of the *C. nankingense* mitogenome in relation to a pair of 681 bp repeats. (**D**) The positive correlation between the frequency of recombination and the length of the medium repeats.

To examine the recombination activity of these repeats (>100 bp), we mapped all the filtered mitochondrial reads (213× coverage and average 31 kb in length) from the ONT sequencing data to the master conformation (MC) and each alternative conformation (AC) derived from these repeats. Although we failed to detect any long reads spanning the whole mitogenome, the conformation of the master molecular structure could be supported by just four reads (Figure 3A). The conflicts between the long reads and the master molecular structure suggested the existence of isomeric and/or subgenomic circles as a consequence of repeat-mediated homologous recombination. We then calculated the proportion of long reads that supported the AC and the MC. A total of five reverse repeats (681, 231, 181, 113, and 103 bp) and two forward repeats (145 and 107 bp) were examined for recombination activity at frequencies ranging from 0.52% to 4.40%, while recombinant forms were not detected for the remaining medium repeats (Table S9). The recombination at the largest repeat (681 bp) was strongly supported by 14 long reads, suggesting that an AC (Figure 3B,C) exists in the *C. nankingense* mitogenome at a frequency of more than 4%. According to our observations, the frequency of recombination and the length of the medium repeats showed an obvious positive correlation (Figure 3D), suggesting that a larger repeat supports a higher frequency of recombination.

### 3.4. The Assemblies of the Asteraceae Mitogenomes

Although two complete mitogenomes from Asteraceae (*H. annuus* and *D. hartwegii*) were available prior to our study, the sequenced mitogenomes remained limited in contrast to the high number of species in this family. Here, we retrieved public genomic Illumina sequencing datasets from 26 Asteraceae species and one Calyceraceae species, *N. patagonicus*, as an outgroup (Table S2). The number of raw reads obtained from the 27 samples ranged from four to 178 million, and the length of the reads ranged from 75 to 250 bp. Based on the optimal assembly method mentioned above for the Illumina-only data, we assembled 27 draft mitogenomes using these data, with the contig number ranging from two to 16 and the sequencing depth ranging from 20× to 1284× (Table S2). The contigs of each species should contain essentially near-complete genetic information of the mitogenomes according to the previous comparisons (Figure 1), although the large plastid-derived fragments and/or one copy of large repeats are absent from the draft mitogenomes (Figure 2). The GC content of the mitogenomes in each species ranged from 44.90% to 45.64%, which is comparable to that of other angiosperms (Table S10), suggesting the validity of each assembly. For the subsequent phylogenetic analysis, the chloroplast candidate contigs, as by-products, were also extracted from the assemblies (Table S2).

### 3.5. Variation in the Gene Content and Constitution in the Asteraceae Mitogenomes

**Figure 4:**
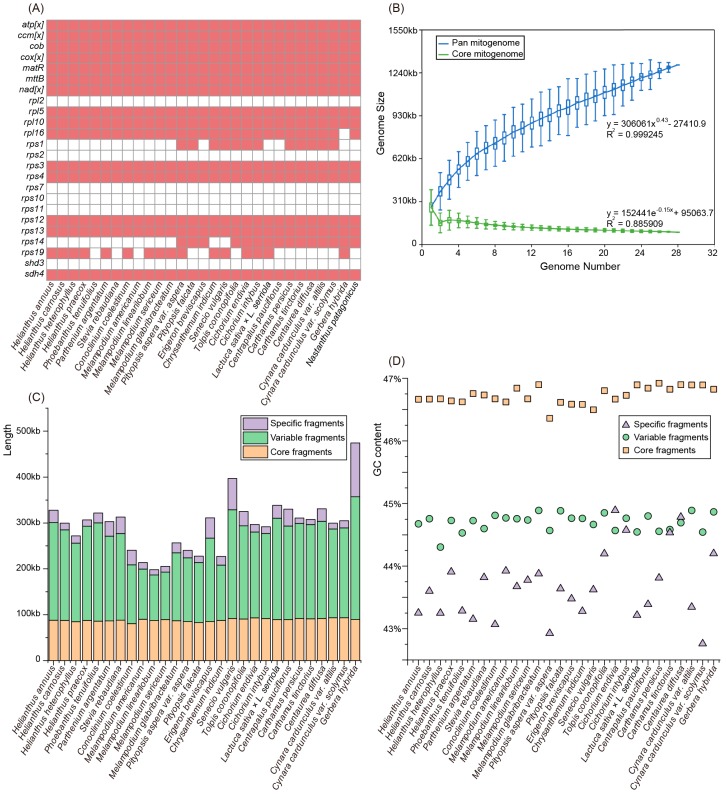
Variation in the gene content and constitution in Asteraceae mitogenomes. (**A**) The gene content of 28 Asteraceae mitogenomes and one Calyceraceae species, *Nastanthus patagonicus*. (**B**) Increase and decrease in the genome size of the pan mitogenome (blue) and core mitogenome (green). (**C**) The size variation of different components in the 28 Asteraceae mitogenomes. (**D**) The GC content variation of different components in the 28 Asteraceae mitogenomes.

Together with the *C. nankingense* mitogenome and previous published *H. annuus* [24] mitogenome, we further performed comparative analysis of mitochondrial gene content and genome size of these 28 Asteraceae taxa. The Asteraceae mitogenomes generally harbored no more than 35 protein-coding genes, of which four genes (*rpl16*, *rps1*, *rps14*, and *rps19*) were not detected in all mitogenomes. Specifically, the *rpl16* gene loss was unique to the *Gerbera hybrida* mitogenome (Figure 4A). Compared with the ancestral gene set of angiosperm mitogenomes, six protein-coding genes (*rpl2*, *rps2*, *rps7*, *rps10*, *rps11*, and *sdh3*) were discarded completely from all the Asteraceae mitogenomes (Figure 4A). Size variability of roughly two-fold was observed among Asteraceae mitogenomes, with a range from 187 kb in Melampodium linearilobum to 357 kb in *G. hybrida* (Table S2). The mitogenomes of Melampodium are generally less than 200 kb in length, which is smaller than the other Asteraceae mitogenomes (Table S2). In particular, only two mitochondrial candidate contigs with a total length of 187 kb were obtained in *M. linearilobum*, which should be the smallest size contig from any non-parasitic angiosperm mitogenome sequenced at present (Table S10).

We used the 28 draft mitogenomes of Asteraceae to perform a pan-mitogenome and mitochondrial constitution analysis. The pan-mitogenome curve indicated an open pan-mitogenome (Figure 4B). This suggests that the dynamic mitogenome experienced substantial exchange of genetic material during evolution. A total of 4448 local collinear blocks (LCBs) were identified from the 28 Asteraceae mitogenomes with a total length of ~1.3 M (pan-genome size), of which 57 LCBs were shared among all these species with an average length of 88 kb (core fragments), representing <50% of each mitogenome (Figure 4C). These core and variable fragments usually contain stable GC contents of 46.7% and 44.7% on average, respectively (Figure 4D). However, the specific fragments have a large variation in size ranging from 11 kb (~3.7% of *Carthamus tinctorius* mitogenome) to 117 kb (~32.8% of *G. hybrida* mitogenome) and variation in GC content ranging from 42.8% (*Cynara cardunculus* var. scolymus) to 44.9% (*Cichorium endivia*) (Figure 4C,D; Table S11). Although the core fragments constitute no more than half of each mitogenome, the length of these fragments (~88 kb) is much longer than the functional regions identified (e.g., 39 kb in the *C. nankingense* mitogenome and 36 kb in the *H. annuus* mitogenome; Table S4), which suggests that more primary functional regions are not recognized.

### 3.6. Phylogenetic Analysis Comparison

To gain insights into the evolution of Asteraceae mitogenomes, four independent sequence matrixes were constructed based on the LCBs and protein-coding genes regions of both the mitogenomes and plastomes of 28 Asteraceae species and *N. patagonicus* as the outgroup. The LCB alignments of the mitogenomes and plastomes comprised a total of 50,949 and 99,143 characters respectively, of which 1357 (2.66%) and 7918 (7.99%) constituted parsimony-informative characters (PICs) (Table S12). The protein-coding genes alignments of the mitogenomes (31 genes) and plastomes (79 genes) comprised 19,519 and 62,124 characters, of which 486 (2.49%) and 3804 (6.12%) constituted PICs, respectively (Tables S12 and S13).

**Figure 5:**
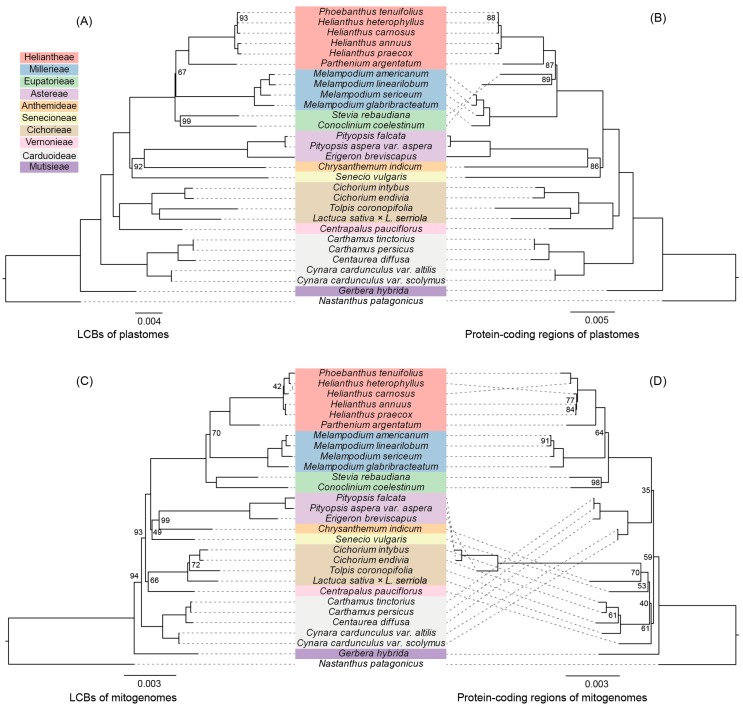
Maximum Likelihood (ML) phylogenetic trees based on different datasets from 28 Asteraceae species and one Calyceraceae species as an outgroup: (**A**) Local collinear blocks (LCB) alignments of the plastomes; (**B**) 79 protein-coding region alignments of the plastomes; (**C**) LCB alignments of the mitogenomes; (**D**) 31 protein-coding region alignments of the mitogenomes. Values indicate the Bootstrap values from the ML analysis. Branches without values indicate maximum support values (100) from all analyses.

The phylogenetic topology obtained from the LCBs of the plastomes was the most highly supported (Figure 5A), and thus we selected this topology to make comparisons with the topologies from other datasets. The topology obtained from the LCBs of the mitogenomes was highly consistent with that obtained from the LCBs of the plastomes, excluding one incongruence located towards the tips of the tree (*H. heterophyllus* and *H. carnosus*; Figure 5C). In addition, the phylogenetic trees from the plastome datasets (LCBs and protein-coding regions) were concordant, with the exception of the *Heliantheae alliance* clade (Heliantheae, Eupatorieae, and Millerieae), which was poorly supported in all of the trees (Figure 5). The phylogeny based on 31 mitochondrial genes did not recover a reliable phylogenetic relationship (Figure 5D). Instead, the phylogenetic topologies based on the other three datasets indicated 10 well-supported tribes, excluding the Heliantheae alliance (Figure 5A–C). Within the Heliantheae alliance, Millerieae was supported by the plastid gene dataset as sister to Eupatorieae plus Heliantheae, while other datasets supported Eupatorieae as sister to Millerieae plus Heliantheae. In addition, Anthemideae is sister to Astereae, while Senecioneae is the next lineage to the Anthemideae. The tribes of Astereae, Anthemideae, and Senecioneae are together sister to the Heliantheae alliance. The tribes of Cichorieae and Vernonieae are then sister to this aforementioned group. Carduoideae is an earlier diverging lineage followed by Mutisieae, which is sister to all other lineages in our research. Overall, in contrast to the phylogeny derived from the 31 mitochondrial genes, the datasets from the LCBs of the mitogenomes recovered a strongly supported phylogenetic topology that was in accordance with the topologies from the plastome datasets, reflecting their potential use for phylogenetic analysis in Asteraceae (Figure 5C).

## 4. Discussion

### 4.1. The Effectiveness of Oxford Nanopore Technology in Mitogenome Research

The greatest hurdle in the research of angiosperm mitogenomes is the difficulty in obtaining complete sequences using traditional NGS technology, which usually generates short reads (<500 bp). The emergence of long-read sequencing technologies (e.g., ONT and PacBio), however, has made it possible to obtain a complex mitogenome as well as investigate both the size and structural variation in vivo. In this study, we investigated the efficacy of ONT sequencing reads for recovering the entire mitogenome of *C. nankingense*. Although we did not obtain any single long read covering the entire mitogenome (208 kb), we found that just four long reads could support the MC (Figure 3A). The longest read belonging to the mitogenome was ~88 kb in length, which indicates that the super long reads can provide stable scaffolds in the assembly of other complex mitogenomes, such as *S. noctiflora* [54] and *Amborella* [55], and can easily span any large repeats and plastid-derived fragments found in other plant mitogenomes.

Compared with the other assembly methods, the hybrid assembly method, using both the ONT and Illumina sequencing data, revealed a great advantage in obtaining a complete plant mitogenome from the genomic DNA data (Figure 1). The assembly from the ONT-only data shows plenty of single base deletions, which required further polishing using the high-quality NGS data. A variety of assembly strategies from the Illumina-only data still play an important role in plant mitogenome sequencing [25,56,57]. The main current NGS technologies also provide plenty unexploited plant mitogenome information. However, the mitogenome assemblies from the Illumina-only data frequently generate a dozen candidate contigs (Table S2), which require further connection based on polymerase chain reaction (PCR) and Sanger sequencing or large-insert mate-paired libraries to provide additional structural information on large repeats and plastid-derived regions. Notably, these contigs should contain near-complete sequence information of the mitogenomes, excluding the large repeats and plastid-derived regions (Figure 2). With the aid of newer long reads technologies, more complex plant mitogenomes should be effortless to recover from genomic DNA using a hybrid assembly strategy.

Oxford Nanopore Technology long reads provide a means of precisely investigating repeat-mediated homologous recombination in plant mitogenomes. The frequency of repeat-mediated recombination is highly variable among different angiosperm mitogenomes [58]. In this study, we investigated structural variation as the consequence of repeat-mediated recombination in the *C. nankingense* mitogenome. We identified a total of seven intermediate repeats related to homologous recombination, which supports the hypothesis of larger size repeats corresponding to increased recombination frequency [59]. We detected an intermediate repeat-mediated recombination (681 bp) at a frequency of up to 4.40%, which is higher than that (1.28%) of the *Nymphaea colorata* mitogenome using the long reads data as well [22]. The mitogenome of the parasitic plant *Viscum scurruloideum* has an almost balanced recombinational frequency (~1:1 stoichiometry) for its four medium repeats (387–593 bp) [7]. However, two large repeats (3.3 kb and 1.5 kb) in the *N. colorata* mitogenome show low recombination frequencies at only 0.24% and 8.18% [22]. It should be mentioned that the repeat-mediated rearrangements in mitogenome could be depending on the developmental stage or some environmental conditions [60] and controlled by the nuclear genes [59]. Repeat-mediated homologous recombination could generate isomeric or subgenomic forms and extensive genomic variation even within the same species [16]. Although a relatively low frequency of homologous recombination was detected in the *C. nankingense* mitogenome, the alternative low-frequency genome conformations (“sublimons”) have the ability to undergo occasional rapid changes in frequency (e.g., substoichiometric shifting) to generate a new MC [61].

### 4.2. The Diversity and Evolution of Asteraceae Mitogenomes

The angiosperm mitogenomes indicate great variations in genomic structure, gene content, and constitution [14]. Here, we compared the gene content and constitution of mitogenomes in 28 Asteraceae species. The vast majority of the variation in mitogenome size could be explained by the differences in intergenic regions [14] rather than the variable gene content (Figure 4A). Simultaneously, there is no doubt that the diversity in mitogenome size could also be attributed to the presence of sizeable repetitive and foreign fragments [2,9]. However, regardless of these contributions, the size of the Asteraceae mitogenomes is still highly variable (Table S2). We aligned 28 Asteraceae mitogenomes to distinguish the core fragments, variable fragments, and specific fragments. The relatively large mitogenomes (*G. hybrida* and *S. vulgaris*) always possessed larger variable fragments (Figure 4C). Compared to other fragments, the specific fragments indicated relatively low and variable GC content, which suggests that these fragments should contain some foreign genetic materials with lower GC content (e.g., plastome and nuclear genome). Generally, the specific fragments are present in each mitogenome, suggesting substantial genetic capture from the plastome, nucleus, or foreign organisms. The diversity in size of the variable fragments could be explained by the genetic escape from the common ancestor of two or more species. Although we failed to precisely identify the intrinsic and extrinsic sources of the variable and specific fragments, these results imply that the substantial genetic exchange of mitogenomes makes large contributions to the diversity of mitogenome constitution.

Compared with plastomes and nuclear genes, mitogenomes are seldom used to reconstruct phylogenies partly due to the slowness in nucleotide substitution rate and the difficulty in complete assembly and direct alignment [3,62]. Here, we identified the LCBs of the mitochondrial contigs from 28 Asteraceae species and one outgroup based on the assembly method mentioned above and performed LCB alignment, which provided an approach for aligning the dynamic mitogenomes. The nuclear phylogenies of 18 tribes in Asteraceae have been well established based on the transcriptome datasets [63]. In this study, based on four independent datasets from the LCBs and protein-coding genes regions of the mitogenomes and plastomes, we reconstructed the phylogenetic relationships of 10 tribes in Asteraceae, of which three tribes (Millerieae, Anthemideae, and Vernonieae) have not been included in previous nuclear phylogenies [63]. The high consistency of the phylogenetic topologies obtained from the LCBs of the mitogenomes and plastomes suggests that the LCB markers of the mitogenomes are effective in resolving relationships in Asteraceae tribes. However, the phylogenetic topology obtained from protein-coding regions of the mitogenomes is inconsistent with other topologies with low support (BS < 80%), which could be attributed to a lack of sufficient PICs (Table S12), or other reasons (e.g., incomplete lineage sorting and cytoplasmic introgression) [64,65]. In conclusion, our analyses based on the core fragments of mitogenomes were able to resolve broad-level relationships within Asteraceae.

## Supplementary Materials

The following are available online http://www.mdpi.com/2073-4425/9/11/547/s1, Table S1: The sequencing data used for different assemblies of the *C. nankingense* mitogenome, Table S2: Information on the mitogenomes of 28 Asteraceae species and one Calyceraceae species, Table S3: Gene content of *C. nankingense* and closely related *H. annuus* mitogenomes, Table S4: Summary of the *C. nankingense* and *H. annuus* mitogenomes, Table S5: Inferred plastid-derived sequences in the mitogenome of *C. nankingense*, Table S6: The identified ORFs in the *C. nankingense* mitogenome, Table S7: The repeats in the *C. nankingense* mitogenome, Table S8: Repeat content among the mitogenome of *C. nankingense*, Table S9: Recombination frequency of the mitogenome in relation to medium repeats, Table S10: Statistics of the sequenced land plant complete mitogenomes, Table S11: The mitochondrial size and GC content variation in 28 Asteraceae mitogenomes, Table S12: Descriptive statistics of the obtained matrices, Table S13: The lengths of the genes used for phylogenetic analysis of 28 Asteraceae species and one Calyceraceae species.
